# Comparison of the Index of Nutritional Quality in Breast Cancer Patients With Healthy Women

**DOI:** 10.3389/fnut.2022.811827

**Published:** 2022-03-24

**Authors:** Mojgan Behrad Nasab, Maryam Afsharfar, Mina Ahmadzadeh, Farhad Vahid, Maryam Gholamalizadeh, Saheb Abbastorki, Sayed Hossein Davoodi, Nazanin Majidi, Mohammad Esmail Akbari, Saeid Doaei

**Affiliations:** ^1^Department of Physical Education and Sport Sciences, Faculty of Sport Science, Central Tehran Branch, Islamic Azad University, Tehran, Iran; ^2^Department of Nutrition, School of Medicine, Zahedan University of Medical Sciences, Zahedan, Iran; ^3^Department of Clinical Nutrition and Dietetics, Faculty of Nutrition and Food Technology, National Nutrition and Food Technology Research Institute, Shahid Beheshti University of Medical Sciences, Tehran, Iran; ^4^Population Health Department, Nutrition and Health Research Group, Luxembourg Institute of Health, Strassen, Luxembourg; ^5^Cancer Research Center, Shahid Beheshti University of Medical Sciences, Tehran, Iran; ^6^Department of Nutrition, Faculty of Nutrition Sciences, Shiraz University of Medical Sciences, Shiraz, Iran; ^7^Department of Nutrition, Science and Research Branch, Islamic Azad University, Tehran, Iran; ^8^Reproductive Health Research Center, Department of Obstetrics and Gynecology, School of Medicine, Al-Zahra Hospital, Guilan University of Medical Sciences, Rasht, Iran

**Keywords:** breast cancer, dietary intake, the index of nutritional quality, cancer, breast

## Abstract

**Background:**

The index of nutritional quality (INQ) is derived from the food frequency questionnaire (FFQ) and is a method of quantitative and qualitative analysis of diet. This study aimed to compare the INQ for different dietary components between breast cancer (BC) patients and healthy control.

**Methods:**

This case-control study was performed on 180 women with BC and 360 healthy women. Data on general characteristics, medical history, anthropometric indices, physical activity, alcohol consumption, reproductive history, smoking, and dietary intake were collected. A valid FFQ was used to assess the intake of nutrients and the INQ was calculated based on the daily intake of the nutrients.

**Results:**

There was a significant association between BC and INQ of vitamin A (OR = 0.07, 0.01–0.29), vitamin E (OR = 0.43, 0.20–0.93), vitamin B6 (OR = 0.003, 0.000–0.021), riboflavin (OR = 0.25, 0.11–0.59), vitamin K (OR = 0.58, 0.37–0.90), biotin (OR = 0.07, 0.02–0.26), vitamin B12 (OR = 0.32, 0.18–0.56), vitamin C (OR = 0.72, 0.55–0.95), zinc (OR = 0.020, 0.005–0.083), calcium (OR = 0.14, 0.04–0.54) and magnesium (OR = 0.003, 0.000–0.024). Further adjustment for BMI disappeared the association between INQ of vitamin C and BC. The results did not change after further adjustments for waist circumstance and total calorie intake

**Conclusion:**

A significant association was observed between BC and the INQ of vitamin A, vitamin E, vitamin B6, riboflavin, vitamin K, biotin, vitamin B12, vitamin C, zinc, calcium, and magnesium. The INQ can be used as an indicator in assessing clinical nutrition-related problems. Future longitudinal studies are needed to confirm these results.

## Introduction

BC is the most frequent cancer among women with an estimated. 2 million new cancer cases diagnosed in 2018 (23% of all cancers) and ranks second overall (10.9% of all cancers). Up to 15 million people were diagnosed with BC by 2020 (1). BC accounts for 23% of all gynecological cancers worldwide (2) and a recent study indicated that incidence and mortality rates of breast cancer are rising (3). In 2008, there were 8 million deaths from malignant diseases, which is estimated to reach 11 million by 2030 (4). BC is influenced by genetics, lifestyle, and environmental factors (1, 2, 4).

Lifestyle including physical activity and nutrition play an important role in cancer. Overweight and obesity were reported to be associated with a higher risk of BC in postmenopausal women (5–7). Higher intakes of saturated fatty acids (8) and alcohol consumption (5, 9) are reported to be associated with an increased risk of BC. On the other hand, a low-fat diet was associated with a 9% reduction in the risk of BC (1). The Mediterranean diet, which is low in red meat and high in fruits and vegetables, is associated with a moderate reduction in the risk of BC in postmenopausal women (10–12).

Some studies reported the positive effect of diets rich in antioxidants, including vitamin E, vitamin A, beta-carotene, vitamin C, folate, unsaturated fatty acids, carbohydrates, vitamin D, carotenoids, phytoestrogens, and fiber on BC. Women who received a healthier diet including antioxidants and low-fat milk were less likely to develop BC than women with a higher intake of fat and red meat (5, 13, 14). A healthy eating pattern with plenty of unrefined grains, vegetables, fruits, nuts, and olive oil and moderate to low intake of saturated fatty acids and red meat may improve overall survival after BC diagnosis. An improper and unbalanced diet increases the risk of BC and nutritional intervention in patients with BC may be an integral part of the treatment approach. Nutritional counseling and supplements may be helpful in reducing BC development (15). However, some studies found no association between nutritional status and BC (16).

Many studies were carried out on the association of dietary components and BC. However, the effect of dietary components in comparison with the recommended dietary allowance (RDA) on the BC risk is not clear. Nutritional quality of diet plays an important role in controlling BC. (INQ) provides a comprehensive list of nutritional components and is a way to qualitatively analyze individual foods, meals, and diets (14, 17). The INQ is derived from a food frequency questionnaire (FFQ) that reflects the frequency of foods received in the past year (18). Few studies with small sample sizes and without adjusting calorie intake were done on the association of INQ and BC (14, 19). So, the aim of this study was to compare the INQ between women with BC and healthy women.

## Methods

### Participants

This case-control study was performed in September 2020 on 180 women with BC and 360 healthy age-matched women referred to the cancer clinic of Shohadaye Tajrish Hospital in Tehran, Iran. Inclusion criteria for the case group were women with BC, age between 35 and 65 years, no more than 1 month after diagnosis of BC, no diseases affecting food intake, and no antioxidant supplements intake. Inclusion criteria in the control group were aged between 35 and 65 years, no more than 1 month after the first diagnosis of BC (regardless of severity and stage of BC), no disease affecting food intake, no use of antioxidant supplements and be Do not have any cancer. Exclusion criteria were the inability to collect the required information and any disease that may affect the diet such as liver disease and diabetes. The written informed consent forms were obtained from all participants prior to the study.

Data on age, demographic characteristics, medical history, daily physical activity (using international physical activity questionnaire), alcohol consumption, reproductive history, smoking, and level of education were collected. Anthropometric indices, including weight, height, body mass index (BMI), and waist circumstance (WC) were measured.

### Dietary Intake

A validated semi-quantitative FFQ was used to assess dietary intake over the past year through face-to-face interviews by a trained nutritionist (20). The FFQ can provide useful information about individual food intake over a period of one year (19, 21). All data obtained from FFQ was converted to grams and dietary data were analyzed using Nutritionist IV software.

INQ was used as a tool designed to assess dietary patterns, including an algorithm that represents the properties of micronutrients and macronutrients and shows the weight coefficients of the epidemiological relationship between nutrients and health outcomes. The components of the algorithm indicated the overall quality of nutrition as follows: Nutrients including vitamin A, vitamin D, vitamin C, vitamin E, vitamin B12, vitamin B6, potassium, calcium, zinc, omega-3 fatty acids, magnesium, selenium, vitamin B5, biotin, niacin, thiamin, riboflavin, iron, total carotenoids, and total bioflavonoids were included. The denominator of the fraction included saturated fatty acids, trans fatty acids, sodium, sugar, and cholesterol. All the nutrients were weighed according to the effect on health, according to the available data. The correlation between INQ scores and the average ranking of foods was evaluated and its validity was confirmed (22).

The INQ of each nutrient was assessed based on the Recommended Dietary Amount (RDA) or Adequate Nutrition (AI) using the following formula: INQ is equal to the amount of nutrient consumed per 1,000 kcal/RDA or AI of that nutrient per 1,000 kcal. Then, the information obtained from the FFQ was analyzed to calculate the average daily consumption of energy and nutrients, and the INQ was calculated based on the daily nutrient intake (23).

### Statistical Analysis

Independent *t*-test and Chi-square methods were used to compare the quantitative and qualitative variables between the two groups, respectively. Logistic regression was used to investigate the relationship between dietary antioxidant index and BC after adjusting for age (model 1), age and BMI (model 2), and age, BMI, WC, and total energy intake (model 3). First, the FFQ Half was completed through interviews. All statistical analyses were performed using SPSS software (version 21) and *P* < 0.05 was considered significant.

## Results

Significant differences were found between healthy women and women with BC in body mass index (27.2 ± 4.4 vs. 29.2 ± 4.2 m/h^2^, *p* = 0.001), pregnancy numbers (4 ± 1.9 vs. 3 ± 1.8, *p* = 0.001), and breastfeeding weeks (59.7 ± 33.5 vs. 33.8 ± 29.4, *p* = 0.001) (Table 1).

**Table 1:** Distribution of characteristics across cases and controls (*n* = 540).

	**Controls (*n =* 360)**		**cases (*n =* 180)**		***P-*Value**
	**Mean ±SD**	**Min–max**	**Mean ±SD**	**Min–max**	
Age (year)	58.3 ± 9.7	33–99	49.9 ± 9.4	38–99	0.27
Body mass index (BMI)	27.2 ± 4.4	20.5–43.1	29.2 ± 4.2	17.3–41.7	0.001
Pregnancy numbers	3.7 ± 1.9	0–12	2.9 ± 1.8	0–8	0.001
Abortion numbers	0.45 ± 0.70	0–4	0.38 ± 0.73	0–3	0.32
Breastfeeding duration	59.7 ± 33.5	0–188	33.8 ± 29.4	0–126	0.001
Menopause age	47.1 ± 5.8	30–59	47.3 ± 5.8	38–55	0.88

Regarding to dietary intake, women with BC had higher intake of calorie (2737 ± 925.3 vs. 2315 ± 1066 Kcal/d, *p* = 0.01), carbohydrate (402.1 ± 124.6 vs. 311.7 ± 170.2 g/d, *p* = 0.001), iron (19.7 ± 6.4 vs. 15.4 ± 12.1 mg/d, *p* = 0.01), thiamin (2.3 ± 0.9 vs. 1.5 ± 0.7 mg/d, *p* = 0.001), niacin (24.3 ± 7.9 vs. 18.2 ± 9.2 mg/d, *p* = 0.001), acid folic (673.5 ± 205.2 vs. 465.4 ± 308.7 μg/d, *p* = 0.001), and selenium (98.7 ± 40.8 vs. 82.6 ± 41.7 μg/d, *p* = 0.01), and lower intake of vitamin D (1.05 ± 0.84 vs. 1.79 ± 1.56 μg/d, *p* = 0.001) (Table 2).

**Table 2:** Dietary intake of breast cancer patients and control groups.

	**Cases (*n =* 180)**		**Controls (*n =* 360)**		***P-*Value**
	**Mean ±SD**	**Min–max**	**Mean ±SD**	**Min–max**	
Total energy intake (Kcal/day)	2737 ± 925.3	963.3–7684	2315 ± 1066	946.2–4989	0.011
Protein (gram/day)	86.6 ± 41.5	36.5–370.4	84.9 ± 41.7	31.2–255.2	0.80
Carbohydrate (gram/day)	402.1 ± 124.6	119.0–792.6	311.7 ± 170.2	100.8–776.6	0.001
Total fat (gram/day)	92.9 ± 42.2	38.9–325.8	93.3 ± 52.8	27.7–244.7	0.95
Cholesterol intake (milligram/day)	242.1 ± 130.1	59.4–895.5	232.7 ± 161.8	13.2–1124	0.70
Saturate fatty acid (gram/day)	29.3 ± 18.9	10.1–169.0	25.2 ± 13.0	9.1–78.9	0.14
MUFA (gram/day)	30.5 ± 13.3	12.5–78.9	35.1 ± 24.5	9.1–152.7	0.15
PUFA (gram/day)	19.4 ± 8.5	7.3–47.9	19.0 ± 12.3	4.6–60.4	0.82
Sodium (milligram/day)	5661 ± 2559	1688–15375	4935 ± 4288	300.5–21467	0.20
Potassium (milligram/day)	4083 ± 1829	1120–14726	4635 ± 4909	1115–38778	0.34
Vitamin A (microgram/day)	485.1 ± 265.0	105.7–1696	876.8 ± 1950	77.5–15636	0.11
Beta-carotene (microgram/day)	3116 ± 1967	644.8–12144	7271 ± 23168	220.6–184065	0.15
Vitamin C (milligram/day)	150.7 ± 113.9	18.9–938.5	217.9 ± 278.6	11.1–1649	0.07
Calcium (milligram/day)	1277 ± 1011	427.8–9467	1198 ± 674.1	94.1–4462	0.56
Iron (milligram/day)	19.7 ± 6.4	5.9–41.5	15.4 ± 12.1	3.6–83.0	0.011
Vitamin D (microgram/day)	1.05 ± 0.84	0.01–3.5	1.79 ± 1.56	0.11–7.6	0.001
Vitamin E (milligram/day)	17.7 ± 11.4	5.3–66.2	17.4 ± 11.6	3.9–82.2	0.91
Thiamin (milligram/day)	2.3 ± 0.9	0.7–6.8	1.5 ± 0.7	0.3–4.2	0.001
Riboflavin (milligram/day)	2.3 ± 1.4	0.9–12.3	2.1 ± 1.3	0.5–10.5	0.57
Niacin (milligram/day)	24.3 ± 7.9	9.9–51.8	18.2 ± 9.2	4.9–44.6	0.001
Pyridoxine (milligram/day)	1.8 ± 0.7	0.8–6.7	2.1 ± 1.2	0.6–7.9	0.11
Acid folic (microgram/day)	673.5 ± 205.2	230.1–1289	465.4 ± 308.7	166.5–1782	0.001
Vitamin B12 (microgram/day)	4.0 ± 3.7	0.6–32.8	4.6 ± 3.1	0.7–17.5	0.19
Zinc (milligram/day)	11.3 ± 6.0	4.1–44.5	12.8 ± 6.9	4.7–35.5	0.18
Manganese (milligram/day)	5.7 ± 2.6	2.1–23.1	4.9 ± 3.7	1.1–25.1	0.09
Selenium (microgram/day)	98.7 ± 40.8	28.4–283.4	82.6 ± 41.7	25.9–300.6	0.01

Regarding to the INQ, women with BC had higher intake of iron (0.82 ± 0.16 vs. 0.71 ± 0.30 mg/d, *p* < 0.01), thiamine (1.57 ± 0.34 vs.1.25 ± 0.35 mg/d, *p* < 0.01), niacin (1.29 ± 0.26 vs.1.15 ± 0.34 mg/d, *p* < 0.01) and folate (1.25 ± 0.25 vs. 0.97 ± 0.29 μg/d, *p* < 0.01), and lower intake of vitamin A (0.45 ± 0.20 vs. 0.87 ± 1.41 μg/d, *p* = 0.02), vitamin C (1.41 ± 0.93 vs. 2.11 ± 2.12 mg/d, *p* < 0.01), vitamin D (0.05 ± 0.04 vs. 0.11 ± 0.08 μg/d, *p* < 0.01), vitamin E (0.86 ± 0.44 vs. 1.03 ± 0.45 mg/d, *p* = 0.03), riboflavin (1.49 ± 0.29 vs. 1.79 ± 0.72 mg/d, *p* < 0.01), vitamin B5 (0.76 ± 0.16 vs. 1.05 ± 0.25 mg/d, *p* < 0.01), biotin (0.75 ± 0.22 vs. 0.96 ± 0.34 mg/d, *p* < 0.01), vitamin B6 (0.88 ± 0.15 vs. 1.22±0.38 mg/d, *p* < 0.01), vitamin B12 (1.13 ± 0.55 vs. 1.91 ± 1.61 μg/d, *p* < 0.01), magnesium (0.86 ± 0.15 vs. 1.17 ± 0.57 mg/d, *p* < 0.01), zinc (1.01 ± 0.25 vs. 1.40 ± 0.45 mg/d, *p* < 0.01), calcium (0.81 ± 0.22 vs. 0.97 ± 0.38 mg/d, *p* < 0.01) (Table 3).

**Table 3:** Comparison of the index of nutritional quality (INQ) of breast cancer patients and control group.

	**Cases (*n =* 180)**	**Controls (*n =* 360)**	***P-*value**
	**Mean±SD**		
Vitamin A	0.45 ± 0.20	0.87 ± 1.41	0.02
VitC (mg)	1.41 ± 0.93	2.11 ± 2.12	<0.01
Fe (mg)	0.82 ± 0.16	0.71 ± 0.30	<0.01
VitD	0.05 ± 0.04	0.11 ± 0.08	<0.01
VitE (mg)	0.86 ± 0.44	1.03 ± 0.45	0.03
VitK	1.03 ± 0.42	3.07 ± 2.89	0.15
Thiamin (mg)	1.57 ± 0.34	1.25 ± 0.35	<0.01
Riboflavin (mg)	1.49 ± 0.29	1.79 ± 0.72	<0.01
Niacin	1.29 ± 0.26	1.15 ± 0.34	<0.01
B5	0.76 ± 0.16	1.05 ± 0.25	<0.01
Biotin	0.75 ± 0.22	0.96 ± 0.34	<0.01
VitB6 (mg)	0.88 ± 0.15	1.22 ± 0.38	<0.01
Folate (mcg)	1.25 ± 0.25	0.97 ± 0.29	<0.01
VitB12 (mcg)	1.13 ± 0.55	1.91 ± 1.61	<0.01
Magnesium (mg)	0.86 ± 0.15	1.17 ± 0.57	<0.01
Zinc (mg)	1.01 ± 0.25	1.40 ± 0.45	<0.01
Calcium (mg)	0.81 ± 0.22	0.97 ± 0.38	<0.01
Selenium (mcg)	1.31 ± 0.33	1.39 ± 0.58	0.34
Cu (mg)	0.47 ± 0.08	0.50 ± 0.23	0.31
Mn (mg)	2.41 ± 0.90	2.37 ± 1.19	0.85

There were significant negative associations between BC and INQ of vitamin A (OR = 0.07, *p* < 0.01), vitamin E (OR = 0.43, *p* = 0.03), vitamin B6 (OR = 0.003, *p* < 0.01), riboflavin (OR = 0.25, *p* < 0.01), vitamin K (OR = 0.58, *p* < 0.01), biotin (OR = 0.07, *p* < 0.01), vitamin B12 (OR = 0.32, *p* < 0.01), vitamin C (OR = 0.72, p = 0.02), zinc (OR = 0.020, *p* < 0.01), calcium (OR = 0.14, *p* < 0.01) and magnesium (OR = 0.003, *p* < 0.01). The association between INQ of vitamin C and BC was disappeared after adjustment for BMI. The results did not change after further adjustments for WC and total energy intake (Table 4).

**Table 4:** The association between breast cancer and the index of nutritional quality (INQ).

	**ORs and 95%CI**	***P-*value^a^**	**ORs and 95%CI**	***P-*value^b^**	**ORs and 95%CI**	***P-*value^f^**
INQ vitamin A	0.07 (0.01–0.29)	<0.01	0.06 (0.01–0.33)	<0.01	0.07 (0.01–0.35)	<0.01
INQ vitamin E	0.43 (0.20–0.93)	0.03	0.35 (0.14–0.84)	0.02	0.36 (0.14–0.87)	0.02
INQ vitamin B6	0.003 (0.000–0.021)	<0.01	0.005 (0.001–0.045)	<0.01	0.005 (0.001–0.046)	<0.01
INQ riboflavin	0.25 (0.11–0.59)	<0.01	0.28 (0.10–0.75)	0.01	0.29 (0.11–0.78)	<0.01
INQ vitamin K	0.58 (0.37–0.90)	<0.01	0.61 (0.39–0.95)	0.03	0.62 (0.40–0.97)	0.04
INQ biotin	0.07 (0.02–0.26)	<0.01	0.10 (0.02–0.47)	<0.01	0.11 (0.02–0.49)	<0.01
INQ vitamin B12	0.32 (0.18–0.56)	<0.01	0.33 (0.17–0.64)	<0.01	0.34 (0.17–0.66)	<0.01
INQ vitamin C	0.72 (0.55–0.95)	0.02	0.78 (0.58–1.04)	0.09	0.77 (0.58–1.03)	0.08
INQ zinc	0.020 (0.005–0.083)	<0.01	0.019 (0.003–0.098)	<0.01	0.017 (0.003–0.092)	<0.01
INQ selenium	0.69 (0.33–1.41)	0.31	0.68 (0.28–1.61)	0.38	0.74 (0.30–1.80)	0.51
INQ calcium	0.14 (0.04–0.54)	<0.01	0.11 (0.02–0.54)	<0.01	0.11 (0.02–0.54)	<0.01
INQ cupper	0.35 (0.04–2.9)	0.33	0.35 (0.03–4.2)	0.41	0.35 (0.03–3.9)	0.40
INQ magnesium	0.003 (0.000–0.024)	<0.01	0.003 (0.000–0.036)	<0.01	0.003 (0.000–0.033)	<0.01
INQ manganese	1.01 (0.75–1.4)	0.85	1.04 (0.72–1.49)	0.82	1.06 (0.74–1.54)	0.73

a *Age adjusted*.

b *Age and BMI adjusted*.

f *Age, BMI, WC, and total energy intake adjusted*.

## Discussion

In the present study, a negative association was observed between BC and the INQ of vitamin A, vitamin E, vitamin B6, riboflavin, vitamin K, biotin, vitamin B12, vitamin C, zinc, calcium, and magnesium. Few studies were performed on the association between the INQ and different types of cancer. For example, one study found an inverse association between the INQ of vitamin A, vitamin D, and vitamin B6, and gastric cancer (19). The INQ is a simple method that can be used in the clinical evaluation of the dietary intake of the patients (14). Standard tools such as INQ may present a more accurate and functional comparison in the association between diet and health outcomes compared to the traditional assessment of dietary intake.

In the present study, energy and carbohydrate intake were higher in women with BC, which was in line with previous studies reporting that consuming more energy increased the risk of cancer by 60 to 70% (24). Many studies were performed on the association of BC and dietary macronutrients. For example, Seiri et al. reported a significant association between BC with dietary fat intake and animal protein (22). However, another study reported that total fat intake was not associated with BC while consuming more olive oil was associated with a reduced risk of BC (23). Another study found no association between overall carbohydrate intake and BC, but a high intake of sweets in sedentary women increases the risk of disease (25). The effects of different types of macronutrients on cancer risk may vary, with some macronutrients having a protective effect and some acting as a risk factor.

Regarding the association between BC and the intake of vitamins, there was a significant association between INQ of vitamin A, vitamin E, vitamin B6, riboflavin, vitamin K, biotin, vitamin B12, and vitamin C with BC. Different micronutrients play crucial roles in cell homeostasis and metabolism and should be received in the recommended amounts. Several studies reported that high consumption of fruits and vegetables reduces the risk of BC due to their antioxidant contents (25–27). For example, the consumption of tomatoes, which is a rich source of lycopene, beta-carotene, vitamin E, and other carotenoids was reported to reduce BC risk by reducing DNA damage and strengthening the immune system. Flavonoids in fruits and vegetables are capable to lower the risk of estrogen-related BC. In addition, a high intake of vitamin C was associated with a lower prevalence of BC in obese women (14, 25–27). Vitamin E induces cancer cell apoptosis that may have a role in the prevention of BC (28). Vitamin D status is also important for protecting against the progression of BC. The biologically active form of vitamin D interacts with the vitamin D receptor (VDR)coordinates the regulation of cancer cell proliferation, differentiation, and survival. So, vitamin D may act as a therapeutic agent for BC through binding and activating the VDRs (29, 30). The protective effect of high folate intake on the risk of BC is more important in people who have enough vitamin B12. Folate is essential for nucleotide synthesis, DNA and RNA methylation, and the conversion of homocysteine to methionine by methionine synthetase, and the presence of vitamin B12 as a cofactor is essential for these reactions (31). Some studies found that excess folate stimulates existing neoplasms (32). In the present study, women with BC consumed higher folate than healthy women. Vitamin K3 was also reported to act as an anti-cancer agent against BC through the mitochondrial apoptosis pathway (33). In addition, in the present study, a negative association was found between INQ of vitamin B6 and BC. Vitamin B6 plays an important role in amino acid metabolism and reduces chronic inflammation. Vitamin B6 levels decrease with increasing levels of inflammatory markers such as interleukin-6, C-reactive protein, and the alpha tumor necrosis factor which are involved in cancer (19).

Regarding the association between BC and minerals, there was a significant association between BC and the INQ of zinc, calcium, and magnesium. Magnesium is involved in the metabolism of nucleic acids and impaired magnesium homeostasis may induce tumor progression (34). Zinc is vital for cell function and plays an important role in the etiology of cancer. It has many roles in the onset, progression, and termination of cancer. The interaction between zinc transporter and immune function may be a mechanism for the role of zinc in cancer (35). In one study, serum zinc levels were not associated with the severity of cancer, so more studies are required in this regard (36). In another study on BC patients, they had high serum levels of calcium, magnesium, iron, copper, manganese, and low levels of sodium, potassium, and arsenic (37).

Some studies failed to find any association between micronutrients and BC. Lazar et al. reported no significant association between vitamin C, vitamin A, vitamin E, selenium, and zinc with the risk of BC (38). The relationship between micronutrients and cancer may be affected by the amount of intake of that nutrient as well as the ratio between their intakes to the recommended amount of that nutrient. The INQ is a measure that compares the amount of intake with the dietary reference intake (DRI) of that nutrient. Interestingly, in the present study, vitamin D intake was significantly lower in BC patients. While vitamin D-related INQ was not associated with BC. According to the findings of the present study, there was no association between vitamin D and BC considering the currently recommended amounts of vitamin D. Higher amounts of vitamin D may be needed to have a preventative effect on BC.

On the other hand, dietary intake of folate, iron, and selenium was significantly higher in patients with BC compared to the healthy controls. However, the INQ levels of these two nutrients were not associated with BC risk. It can be inferred that considering the intake of nutrients may be misleading. The INQ compares the mean intake with the recommended amounts of the nutrients and can be preferred for a better understanding of the association between health outcomes and dietary components. Few studies were performed on the association between BC and the INQ. Vahid et al. found that there was an inverse relationship between INQs of vitamins A, C, B1, B2, B12 and selenium and BC risk (14) which was partially in line with the present study. However, this study was based on a smaller sample size compared to the present study which may have significantly influenced the association between INQ of some nutrients and BC.

However, this study had some limitations. Biochemical parameters such as Hb, Hct, MCV, MCH were not measured in the present study and the effects of the nutrients on these biomarkers are unknown. In addition, this case-control study was limited to BC patients, which makes it difficult to generalize the results to other cancers. This study focused on INQ, which is calculated based on DRI and cannot be used for nutrients or dietary components without DRI. Future prospective studies with larger sample sizes on different cancers are warranted to evaluation the value of the INQ in determining the role of nutrients in reducing cancer risk. The advantage of using INQ is the adjustment of the amount of nutrients based on the amount of calories received and comparison them with the recommended amount of diet.

## Conclusion

A significant relationship was observed between BC and INQ of vitamin A, vitamin E, vitamin B6, riboflavin, vitamin K, biotin, vitamin B12, vitamin C, zinc, calcium, and magnesium. The INQ can play an important role in the clinical evaluation of the dietary intake of the patients. The use of standard tools and indicators such as INQ compared to the traditional assessment of dietary intake may present a more accurate and functional comparison in the association between diet and different health outcomes. Future longitudinal studies are needed to confirm these results.

## Data Availability Statement

The raw data supporting the conclusions of this article will be made available by the corresponding author, without undue reservation, to any qualified researcher.

## Ethics Statement

The studies involving human participants were reviewed and approved by IR.MEDSAB.REC.1397.070. The patients/participants provided their written informed consent to participate in this study.

## Author Contributions

MG, SDo, and MEA designed the study involved in the data collection, analysis, and drafting of the manuscript. MB, MAf, MAh, FV, SA, SHD, and NM were involved in the design of the study, analysis of the data, and critically reviewed the manuscript. All authors read and approved the final manuscript.

## Funding

Funding for this study was provided by Shahid Beheshti University of Medical Sciences, Tehran, Iran (17260).

## Conflict of Interest

The authors declare that the research was conducted in the absence of any commercial or financial relationships that could be construed as a potential conflict of interest.

## Publisher's Note

All claims expressed in this article are solely those of the authors and do not necessarily represent those of their affiliated organizations, or those of the publisher, the editors and the reviewers. Any product that may be evaluated in this article, or claim that may be made by its manufacturer, is not guaranteed or endorsed by the publisher.

## Abbreviations

FFQ: Food frequency questionnaire
INQ: index of nutritional quality
BC: breast cancer
BMI: body mass index
WC: waist circumstance
RDA: Recommended Dietary Allowance
DRI: Dietary Reference Intake.
